# An integrated machine learning framework for a discriminative analysis of schizophrenia using multi-biological data

**DOI:** 10.1038/s41598-021-94007-9

**Published:** 2021-07-19

**Authors:** Peng-fei Ke, Dong-sheng Xiong, Jia-hui Li, Zhi-lin Pan, Jing Zhou, Shi-jia Li, Jie Song, Xiao-yi Chen, Gui-xiang Li, Jun Chen, Xiao-bo Li, Yu-ping Ning, Feng-chun Wu, Kai Wu

**Affiliations:** 1 Department of Biomedical Engineering, School of Material Science and Engineering, South China University of Technology, Guangzhou, 510006 Guangdong China; 2 The Affiliated Brain Hospital of Guangzhou Medical University, Guangzhou Huiai Hospital, Guangzhou, 510370 Guangdong China; 3 Guangdong Engineering Technology Research Center for Translational Medicine of Mental Disorders, Guangzhou, 510370 China; 4 Guangdong Engineering Technology Research Center for Diagnosis and Rehabilitation of Dementia, Guangzhou, 510500 China; 5 National Engineering Research Center for Tissue Restoration and Reconstruction, South China University of Technology, Guangzhou, 510006 China; 6 Key Laboratory of Biomedical Engineering of Guangdong Province, South China University of Technology, Guangzhou, 510006 China; 7 National Engineering Research Center for Healthcare Devices, Guangzhou, 510500 China; 8 Department of Biomedical Engineering, New Jersey Institute of Technology, Newark, NJ USA; 9 Department of Nuclear Medicine and Radiology, Institute of Development, Aging and Cancer, Tohoku University, Sendai, 980-8575 Japan

**Keywords:** Predictive markers, Psychiatric disorders

## Abstract

Finding effective and objective biomarkers to inform the diagnosis of schizophrenia is of great importance yet remains challenging. Relatively little work has been conducted on multi-biological data for the diagnosis of schizophrenia. In this cross-sectional study, we extracted multiple features from three types of biological data, including gut microbiota data, blood data, and electroencephalogram data. Then, an integrated framework of machine learning consisting of five classifiers, three feature selection algorithms, and four cross validation methods was used to discriminate patients with schizophrenia from healthy controls. Our results show that the support vector machine classifier without feature selection using the input features of multi-biological data achieved the best performance, with an accuracy of 91.7% and an AUC of 96.5% (*p* < 0.05). These results indicate that multi-biological data showed better discriminative capacity for patients with schizophrenia than single biological data. The top 5% discriminative features selected from the optimal model include the gut microbiota features (*Lactobacillus*, *Haemophilus*, and *Prevotella*), the blood features (superoxide dismutase level, monocyte-lymphocyte ratio, and neutrophil count), and the electroencephalogram features (nodal local efficiency, nodal efficiency, and nodal shortest path length in the temporal and frontal-parietal brain areas). The proposed integrated framework may be helpful for understanding the pathophysiology of schizophrenia and developing biomarkers for schizophrenia using multi-biological data.

## Introduction

Finding effective and objective biomarkers to inform the diagnosis of schizophrenia (SZ) is of great importance yet remains challenging^1,2^. Currently, increasing evidence has shown that the gut microbiome, blood and electroencephalogram (EEG) provide abundant clues for the diagnosis of SZ. Recently, several studies have indicated that patients with SZ show an altered gut microbiome composition^3–5^, which is significantly associated with the severity of symptoms^3^ and human brain structure and function^5^. Moreover, a large number of previous studies indicate alterations in both pro- and anti-inflammatory molecules in the central nervous system, which have also been detected in peripheral blood, and may correlate with SZ symptoms^6–8^. Furthermore, several EEG analyses indicate that patients with SZ show significant alterations in the power of various frequency bands, including the increases in delta and theta waves, the decreases in alpha waves and the increases in beta and gamma waves^9–12^. However, most of these alterations are observed at the group level with substantial variability among individuals with the same phenotypic diagnosis. Consequently, none of these alterations has proven to have the ability to reliably aid in the differential diagnosis of SZ to date^1,13^. Therefore, studies analyzing how gut microbiota data, blood data and EEG data behave at an individual level are important; for example, this information could be used to better understand the pathology and identify objective biomarkers for the clinical diagnosis of SZ^14^.

Recently, pattern recognition based on machine learning has attracted increasing attention, which is well suited for the identification of subtle patterns of information in the data and, consequently, is useful to better predict the diagnosis at an individual level^1,15–17^. Using a variety of biological data, such as gut microbiota data^4,18^, blood data^14^, and EEG data^12,19–22^, along with machine learning techniques, hundreds of studies have been performed in an attempt to achieve the accurate classification of patients with SZ. For instance, a previous study^4^ used Boruta variable selection to select the most discriminatory taxa and random forests methods to develop a classifier and predict SZ based on the important microbiota features. A receiver operating characteristic curve analysis revealed that 12 significant microbiota biomarkers were capable of being used as diagnostic factors. A more recent study^14^ developed a probabilistic multi-domain data integration model consisting of immune and inflammatory biomarkers in peripheral blood and cognitive biomarkers using machine learning to discriminate patients with SZ from healthy controls (HCs). Another study^20^ applied the 1-norm support vector machine (SVM) method based on EEG signals of 64 channels during a working memory task to classify patients with SZ versus healthy controls and an accuracy of 87% was achieved. Despite these advances, previous discriminative studies of SZ have primarily focused on biomarkers extracted from a single type of biological data, which only capture partial information about the human body and therefore influence the resulting classification performance. Currently, increasing evidence has shown that the combination of multimodal imaging data can further improve the classification performance^23–26^.

In this study, we collected multi-biological data, including gut microbiota data, blood data and EEG data, from patients with SZ and HCs. An integrated framework of machine learning consisting of multi-biological data, multi-classifiers, multi-feature selection algorithms and multi-cross validation methods, was used to discriminate patients with SZ from HCs. Numerous previous studies have shown that: (1) combining multi-biological data provides more complementary information for discriminative analysis^14,24^; (2) multi-classifiers, multi-feature selection algorithms can better adapt to heterogeneous biological data^27,28^; (3) multi-cross validation methods can test the performance of models more credibly^21^. In this study, we proposed an integrated framework to improve the classification performance and the understanding of biomarker identification for SZ.

## Materials and methods

### Participants

The final sample comprised 99 participants, including 49 patients with SZ and 50 HCs. Patients with SZ were recruited from the Affiliated Brain Hospital of Guangzhou Medical University, Guangzhou, and met the diagnostic criteria in the fourth edition of the Diagnostic and Statistical Manual of Mental Disorder-IV-Text Revision (DSM-IV-TR). The psychopathology and symptom severity of the patients were evaluated with the positive and negative syndrome scale (PANSS) and the psychiatric symptoms were steady for > 2 weeks; the PANSS evaluated the rate of change at ≤ 20% over 2 weeks and the total score on the PANSS was ≥ 30. Patients with SZ were excluded if they met any of the following criteria: (1) any other psychiatric axis I disorder meeting the DSM-IV criteria; (2) constipation, diarrhea, diabetes, hypertension, heart disease, thyroid diseases or any somatic diseases; (3) a history of epilepsy, with the exception of febrile convulsions; (4) a history of having received electroconvulsive therapy in the past 6 months; (5) lactating, pregnant, or planning to become pregnant; (6) alcohol dependence; or (7) noncompliant with drug treatment or a lack of legal guardians.

The HCs were solicited from the local community through advertisements and were screened for their family clinical history and a history of mental illness. All healthy subjects had no history of brain disease (such as pain, schizophrenia, concussion, brain trauma, etc.), ocular disease, treatment with psychotropic medication and drug abuse. In addition, the subjects were asked not to drink alcohol, tea, coffee or any other food or drugs that might excite the central nervous system within 48 h before the experiment and that they get enough sleep the night before the test.

The study protocol was approved by the ethics committees of the Affiliated Brain Hospital of Guangzhou Medical University, and written informed consent was obtained from each subject or their legal guardian prior to the study.

### Multi-biological data acquisition and preprocessing

#### EEG recording and preprocessing

Three minutes of resting EEG data were recorded from 16 scalp electrodes (i.e., Fp1, Fp2, F3, F4, C3, C4, P3, P4, O1, O2, F7, F8, T3, T4, T5, and T6) while the participant’s eyes were closed according to the International 10/20 System and referenced to electrode Cz (UEA-B, symptom, China). All electrode impedances were maintained at less than 10 kΩ. Signals were amplified and digitized using a sampling rate of 1000 Hz and a 60-Hz low-pass filter during recording.

EEG preprocessing was conducted using MATLAB software (Math Works, Natick, MA). Preprocessing was divided into four steps: electrode positioning, filtering, elimination of bad signal segments and signal frequency band decomposition. A bandpass filter of 0.1–45 Hz was used to improve the quality of the signal. Then, the EEG signal was divided into several epochs of 2 s, and artifact noise, such as eye blinks and movement, was removed by technicians. Finally, the signal was divided into seven frequency subbands by a finite impulse response filter: delta band (1.5–4 Hz), theta band (4–8 Hz), alpha1 band (8–10 Hz), alpha2 band (10–13 Hz), beta1 band (13–20 Hz), beta2 band (20–30 Hz) and gamma band (30–45 Hz).

#### Fecal sample collection and preprocessing

Fresh fecal samples were collected from all subjects and then were stored at − 80 °C until DNA extraction. Two hundred milligrams of each fecal sample were used for DNA extraction.

The DNA extraction method was consistent with our previously published report^3^. Sequencing of the V4 region of the 16S rRNA gene was performed on the Illumina MiSeq platform. The row sequences were processed using QIIME2 (version 2018.6). Forward and reverse reads for each individual sample were demultiplexed, joined and quality filtered. We obtained a total of 4,561,105 joined sequences from these raw paired-end sequences, ranging from 15,449 to 95,651, and the average length of all joined sequences was approximately 251 bp. Then, the DADA2^29^ algorithm was used for sequence quality control and feature table construction. After quality filtering, we obtained 4,148,451 high-quality reads, ranging from 13,581 to 90,203 and with a mean of 41,903.5 reads. Then, all the high-quality reads were clustered, 2031 features were obtained, and the frequency per feature ranged from 2 to 533,200, with an average of 3356.9. We used a pretrained naïve Bayes classifier for taxonomic annotation, and this classifier was trained on the Greengenes database (version 13.8). The raw sequence data reported in this article have been deposited in GenBank in the National Center for Biotechnology Information (NCBI) under accession numbers MT545156–MT547172, which are publicly accessible at https://www.ncbi.nlm.nih.gov.

#### Blood collection and preprocessing

Three milliliters of blood were collected from control subjects and patients by simple venipuncture between 7.00 and 9.00 a.m., after an overnight fasting and tobacco abstinence for more than 12 h. Blood biochemical indicators were detected with an automatic biochemical analyzer.

### Multi-biological feature extraction

#### EEG feature extraction

**Figure 1:**
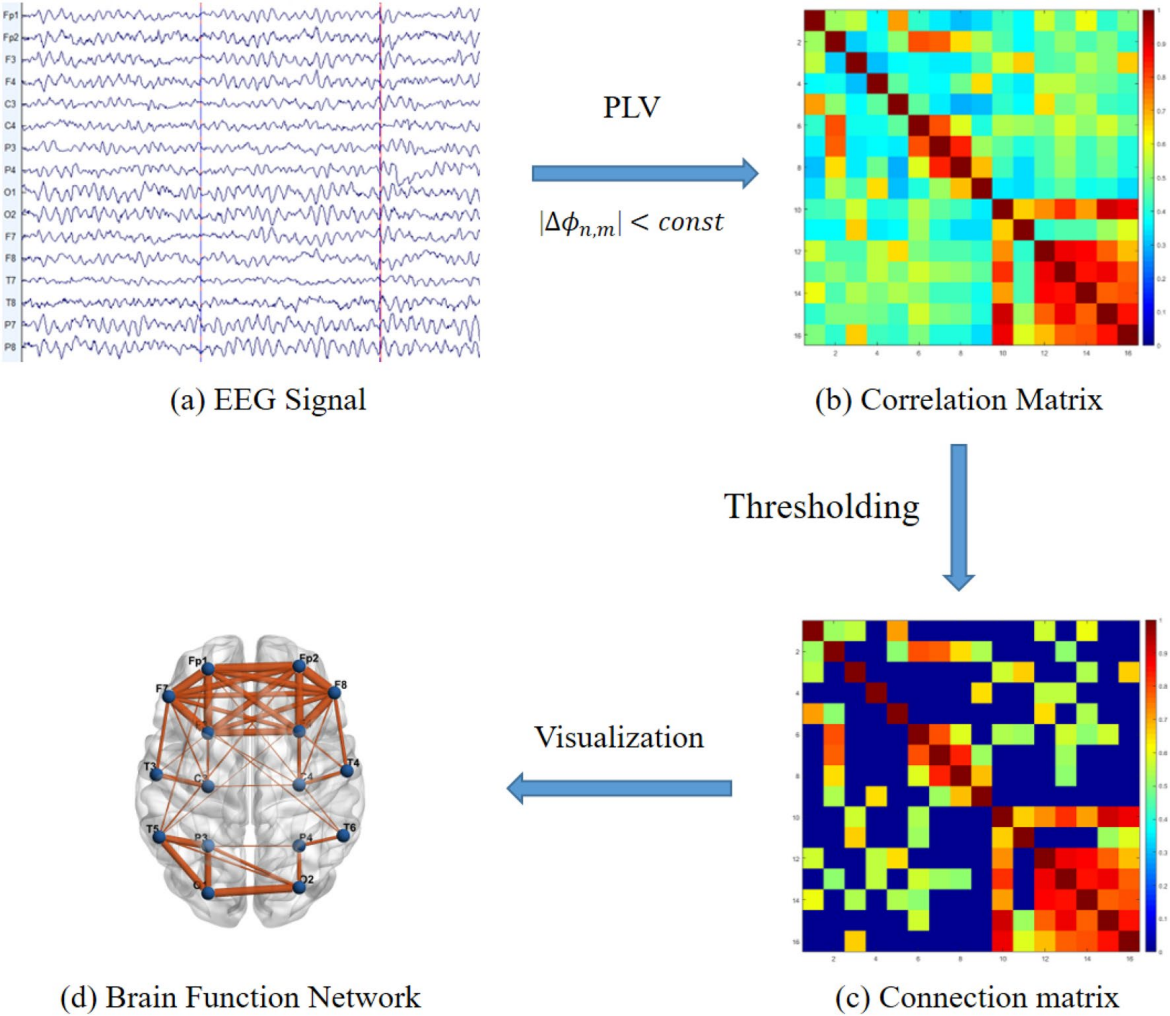
Flow chart of the brain network construction of the EEG signal. *EEG* electroencephalogram, *PLV* phase locking value. Figure (**a**) was generated by an EEG processing tool of “EEGLAB” (Version 2019.0, https://sccn.ucsd.edu/eeglab/index.php), based on MATLAB (Version R2018a). Figure (**b**–**d**) were generated by a brain network visualization tool of "BrainNet Viewer" (Version1.62, https://www.nitrc.org/projects/bnv/), based on MATLAB (Version R2018a).

In this study, we used the phase-locked value (PLV) method to quantify the functional connectivity (FC) between any two channels of EEG signals, as shown in Fig. 1.

The instantaneous phase ${\varnothing }\left(t\right)$ was calculated from the signal $x(t)$ by using the Hilbert transform:

$$\tilde{x }\left(t\right)=h\left(t\right)*x(t)={\int }_{-\infty }^{+\infty }x\left(\tau \right)h(t-\tau )d\tau =\frac{1}{\pi }{\int }_{-\infty }^{+\infty }\frac{x(\tau )}{t-\tau }d\tau $$

The phase was computed using the following expression:

$${\varnothing }_{x}\left(t\right)={\text{arctan}}\frac{\tilde{x }\left(t\right)}{x(t)}$$

Phase synchronization is defined as the locking of phases of two oscillators:

$$\varnothing \left(t\right)={\varnothing }_{x}\left(t\right)-{\varnothing }_{y}\left(t\right)$$

The phase-locking value (PLV) is defined as:

$${\text{R}}=\left|\frac{1}{N}\sum_{j=0}^{N-1}{e}^{i\varnothing (j\Delta t)}\right|$$

where $i$ denotes the imaginary unit, N indicates the total number of samples, and $\Delta t$ is the bespeak time between the successive samples ${\text{j}}$ from 1 to N − 1.

In this study, a cost threshold strategy was used to analyze global and nodal attributes of the functional brain network (FBN). The cost threshold should be greater than $2*{\text{ln}}\left({\text{N}}\right)/N$, where N represented the number of nodes, to ensure that the small-world properties of FBNs were estimable^30^. Moreover, the resulting brain networks should have sparse properties and distinguishable properties compared to the degree-matched random networks. Thus, we selected the small-world regime as a range of cost thresholds ($34\%  \le {\text{cost}} \le 73\% ,\;{\text{step}} = 1\%$). The area under the curve for each attribute was then calculated across the range of cost thresholds and used in a subsequent analysis. Here, all the global and nodal attributes were calculated using the toolbox of BCT^31^. Global attributes include the global clustering coefficient (aCp), shortest path length (aLp), global efficiency (aEg), local efficiency (aEloc), aGamma, aLambda, and aSigma. Nodal attributes include the clustering coefficient (aNCp), nodal shortest path length (aNLp), nodal efficiency (aNe), nodal local efficiency (aNLe), and degree centrality (aDc). In this study, 56 global attributes of an FBN and 640 nodal attributes of 16 nodes were computed from the whole band and seven frequency subbands. Importantly, any features with missing values for any participant were removed. Finally, 48 global attributes and 526 node attributes were used for the subsequent analysis.

#### Gut microbiota feature extraction

Through gene sequencing technology, microbiota markers from 171 species were obtained from all subjects. Among them, any microbiota marker that was missing in more than 85% of the participants was removed. Ninety-four microbiota markers were removed, and 77 gut microbiota markers were selected for the final analysis.

#### Blood feature extraction

The white blood cells (WBC) count, neutrophils (NEU) count, lymphocytes (LYM) count, platelets (PLT) count and monocytes (MON) count were recorded from complete blood counts after routine blood tests. Four blood indicators inflammation and immunity, including the neutrophil–lymphocyte ratio (NLR), platelet–lymphocyte ratio (PLR), monocyte–lymphocyte ratio (MLR) and systemic immune inflammation index (SIII), were calculated based on the numbers of the five cell types described above. Moreover, the oxidative stress indicators, including superoxide dismutase (SOD), homocysteine and C-reactive protein (CRP) levels, were also detected in the collected serum. In conclusion, we collected a total of 12 blood features for the final analysis.

### Statistical analysis

Statistical analyses were conducted using SPSS software version 22 (IBM). The comparison of the sex distribution between the two groups was performed using the *χ*^*2*^ test. Comparisons including age and education years between the two groups were performed using a two-tailed two-sample *t* test. Unless specified otherwise, the significance of all tests was set to *p* < 0.05, or FDR-corrected *p* < 0.05.

### Machine learning

**Figure 2:**
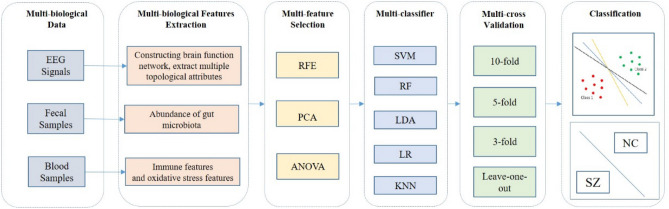
Overview of the proposed integrated machine learning framework for classifying schizophrenia. The proposed integrated machine learning framework for classifying schizophrenia consists of 5 M-methods. (**a**) Multi-biological data were collected from all subjects, including electroencephalogram (EEG) data, fecal data and blood data. (**b**) Multi-biological features were extracted from multi-biological data. (**c**) Multi-feature selection algorithms were used to eliminate redundant features, including recursive feature elimination (RFE), principal component analysis (PCA), and analysis of variance (ANOVA) (**d**) Multi-classifier were used to match heterogeneous biological features including support vector machine (SVM), random forest (RF), linear discriminant analysis (LDA), logistic regression (LR), and k-nearest neighbor (KNN) methods. (**e**) Multi-cross validation methods including tenfold, fivefold, threefold, and leave-one-out methods, were used to evaluate the performance of the trained model.

We developed an integrated framework of machine learning to discriminate patients with SZ from HCs (Fig. 2). Briefly, the framework involved three phases: the data preparation, model training, and independent model testing.

#### Data preparation

Data preparation included feature extraction and subject grouping. We extracted three types of biological features from fecal data, blood data, and EEG data, namely, gut microbiota features, blood features, and EEG features, respectively. For the final analysis, Seventy-seven gut microbiota features, 12 blood features, and 574 EEG features were selected for the final analysis. Three types of biological features were used as input features for machine learning, either individually or in combination, to form four input feature sets. At this stage, we randomly split the set of participants into two groups, a training dataset and an independent testing dataset, at a ratio of 3:1. The training dataset was used to train the model parameters, and the independent testing dataset was used to evaluate the performance of the trained model.

#### Model training

**Figure 3:**
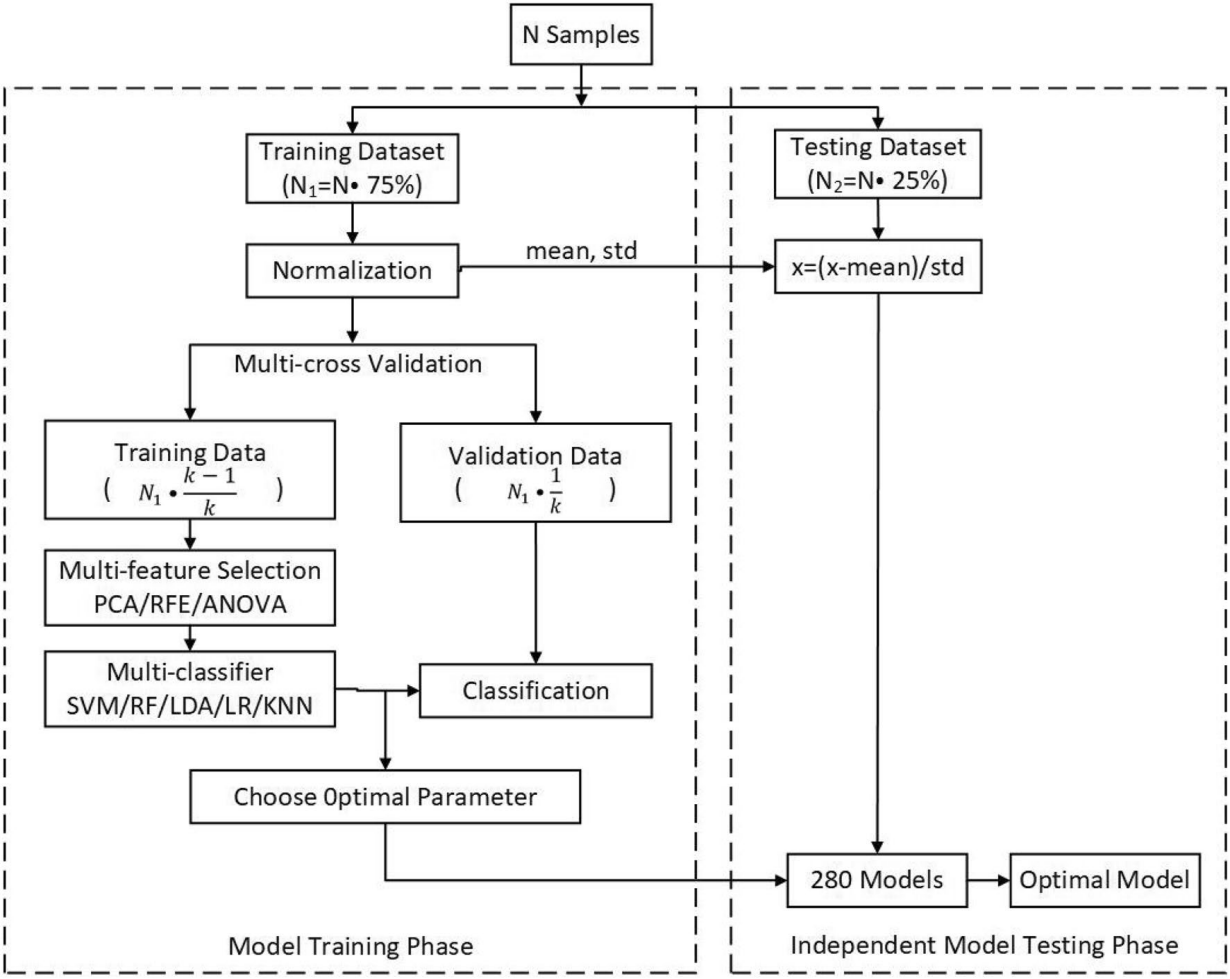
Flowchart of the machine learning classification method.

The specific details of the model training phase and independent model testing phase are shown in Fig. 3. The model training procedures included three steps: multi-feature selection algorithms, multi-classifier, and multi-cross validation methods.

Because some features are less effective, irrelevant, or redundant for classification, and too many features may cause “overfitting”, effective feature selection methods can be used to identify the discriminative features and facilitate disease classification and interpretation. Three feature selection algorithms were used on each classifier, including principal component analysis (PCA), recursive feature elimination (RFE) and analysis of variance (ANOVA), to observe the classification effect of the classifier.

A specific classification model directly based on multi-biological data is difficult to build due to heterogeneity. Therefore, the use of several machine learning methods to construct different classification models is meaningful. In this study, we used five different popular classifiers including support vector machine (SVM), random forest (RF), linear discriminant analysis (LDA), logistic regression (LR) and k-nearest neighbor (KNN), to determine the most suitable model and to evaluate classification performance based on single and combined biological features.

Multi-cross validation methods were used to analyze the training set, including tenfold, fivefold, threefold and leave-one-out methods, and to ensure that the sample size was sufficiently large to train the model and prevent overfitting caused by insufficient training. Several combinations of the aforementioned procedures were investigated for optimized data analysis. PCA and RFE feature selection algorithms were unable to be used due to the small dimension of blood features. As a result, 280 models were obtained based on four input feature sets, five classifiers, three feature selection algorithms and four cross validation methods. Model training in the second phase was performed with their application restricted to the training data set.

#### Independent model testing

In the third phase, we used an independent testing dataset to estimate the generalizability of 280 models arising from the second phase. We utilized the metrics of accuracy, sensitivity and specificity to quantitatively estimate the performance of all the methods mentioned in this study. Moreover, we plotted receiver operating characteristic (ROC) curves and then calculated the area under the curve (AUC) for each classification situation to examine the possibility of correctly discriminating patients with SZ and HCs.

A permutation test was applied to evaluate the statistical significance of the classification results. In our analysis, we disrupted the labels of all samples 1000 times, and the *p* value was computed as the proportion of accuracies that were no less than the accuracy obtained with the original data. The statistical significance was set to *p* < 0.05. All automatic classification work was performed using NEURO-LEARN (https://github.com/Raniac/NEURO-LEARN^32^), which is a solution for collaborative pattern analysis of neuroimaging data.

## Results

### Participants

The resulting data set comprised 99 participants, including 49 patients with SZ (mean [SD] age, 42.06 [12.48] years; 24 [49.0%] males) and 50 HCs (mean [SD] age, 41.70 [13.07] years; 23 [46.0%] males). Significant differences in either age (*t* = 0.141, *p* = 0.888) and sex (*t* = 0.294, *p* = 0.769) were not observed between the patients with SZ and HC group. See Table 1 for a detailed description of other characteristics.

**Table 1:** Demographic and clinical characteristics used in the analysis.

Characteristic	HCs (n = 50)	SZs (n = 49)	*p* value
Age, mean (SD) (years)	41.7 (13.1)	42.1 (12.5)	0.89
**Sex, No (%)**			
Male	23 (46.0)	24 (49.0)	0.77
Female	27 (54.0)	25 (51.0)	
Education years, mean (SD) (years)	14.2 (3.6)	11.6 (3.4)	< 0.001
PANSS, mean (SD)	NA	58.84 (17.50)	NA

*HCs* healthy controls, *SZs* patients with schizophrenia, *NA* not applicable.

### Classification results and analysis

**Figure 4:**
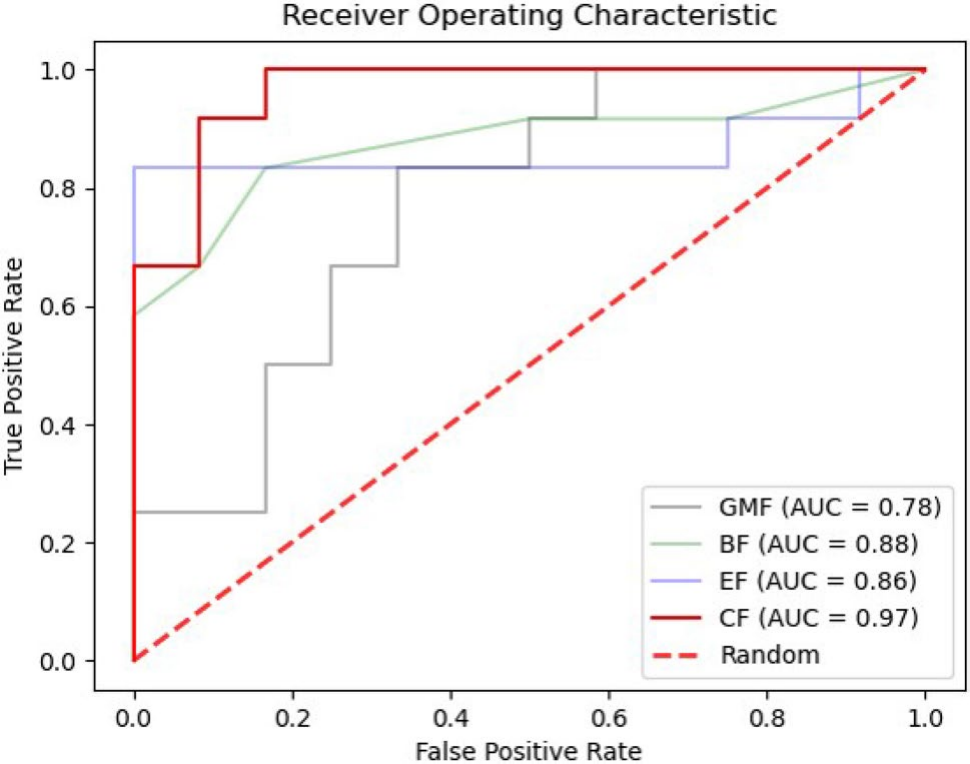
Areas under the receiver operating characteristic curves (AUC) for the best model comparing the gut microbiota features, blood features, electroencephalogram features and the combination of GMV, BF and EF as the input for machine learning. Each curve in the figure represents the ROC curve of the best model using different input features. *GMF* gut microbiota features, *BF* blood features, *EF* electroencephalogram features, *CF* combined features. This figure was generated by “Visual Studio Code” (Version 1.56, https://code.visualstudio.com/).

We used an independent testing dataset to estimate the generalizability of the 280 models. The classification performance of the tenfold cross validation method, fivefold cross validation method, threefold cross validation method, and leave-one-out cross validation method (eTables 1–Table 4 in the “Supplementary S1”) was obtained. No significant differences were observed among the results of multi-cross validation methods. Table 2 shows the classification performance of the model obtained using different input features with tenfold cross validation methods. The optimal classification performance was achieved when multi-biological features were combined as input features, with 91.7% accuracy, 91.7% sensitivity, 91.7% specificity, and 96.5% AUC. The performance of the classifier based on multi-biological features was better than that of the classifiers using a single type of biological feature (Fig. 4). In addition, the blood features achieved the best classification performance when using a single type of biological feature, with an accuracy of 83.3% and an AUC of 87.5%. When gut microbiota features, blood features, and EEG features were used as input feature sets alone, the classifiers and feature selection algorithms of the optimal model were inconsistent, potentially due to the heterogeneity of biological data. The SVM, LR and RF classifiers without using any feature selection algorithm displayed better classification performance when using combined features, with AUCs greater than 90%.

**Table 2:** Classification performance of the optimal model including different input features using the integrated machine learning framework (tenfold).

Input feature	Feature Selection Method	Classifier	Accuracy (%)	Sensitivity (%)	Specificity (%)	AUC	*p *value^a^
Gut microbiota features (n = 77)	RFE	RF	70.8	58.3	83.3	0.80	0.03
Blood features (n = 12)	None^b^	KNN	83.3	83.3	83.3	0.88	0.010
EEG features (n = 574)	RFE	RF	79.2	83.3	75.0	0.90	0.010
Combined features (n = 663)	None	SVM	91.7	91.7	91.7	0.97	0.010

*AUC* area under the receiver operating characteristic curve, *RFE* recursive feature elimination, *KNN* k-nearest neighbor, *LR* logistic regression, *RF* random forest, *SVM* support vector machine, *EEG* electroencephalogram.

^a^The statistical significance of the permutation test was set to *p* < 0.05.

^b^None means no feature selection algorithm was used.

### Discriminative features

In this subsection, the most informative features selected to differentiate the patients with SZ from HCs are reported. We discuss the most discriminative features from the optimal model that were generated when combined features were used. For quantitative analysis, the top 34 (5% of the total number of features) commonly selected features are summarized in Table 3, which shows the top 34 features for classification listed in descending order of their weights, including 14 gut microbiota features, 8 blood features, and 12 EEG features.

**Table 3:** Top 34 features (5%) showing the most discriminative biomarkers for multi-biological predictions.

Number	Feature name	Feature type	Number	Feature name	Feature type
1	SOD	Blood	18	*Undefined*^b^	GM
2	MLR	Blood	19	*Anaerostipes*	GM
3	*Lactobacillus*	GM	20	PLT	Blood
4	MON	Blood	21	alpha2_aNLe_P4	EEG
5	*Haemophilus*	GM	22	*Dialister*	GM
6	*Prevotella*	GM	23	beta1_aLambda	EEG
7	NEU	Blood	24	*Slackia*	GM
8	CRP	Blood	25	*Undefined*	GM
9	*Megamonas*	GM	26	*Odoribacter*	GM
10	theta_aNLe_T6^a^	EEG	27	*Ruminococcus*^c^	GM
11	theta_aNe_T6	EEG	28	theta_aDc_FP1	EEG
12	theta__aNCp_T6	EEG	29	alpha2__aNCp_P4	EEG
13	WBC	Blood	30	beta2_aNLe_FP2	EEG
14	NLR	Blood	31	beta2_aDc_O2	EEG
15	*Collinsella*	GM	32	*Gemmiger*	GM
16	gamma_aDc_F7	EEG	33	alpha2_aNLe_T4	EEG
17	*Clostridium*	GM	34	alpha2__aNCp_T4	EEG

The top 34 features are listed in the descending order of their weights.

*GM* gut microbiota, *EEG* electroencephalogram, *SOD* superoxide dismutase, *MLR* monocyte–lymphocyte ratio, *MON* monocyte, *NEU* neutrophil, *CRP* C-reactive protein, *WBC* white blood cell, *NLR* neutrophil–lymphocyte ratio, *PLT* platelet, *aNLe* nodal local efficiency, *aNe* nodal efficiency, *aNCp* nodal clustering coefficient, *aDc* degree centrality.

^a^The EEG features are represented as a_b_c, where a represents the frequency band, b represents brain network attributes, and c represents the electrode channel.

^b^Undefined *Lachnospiraceae.*

^c^Undefined *Ruminococcaceae.*

## Discussion

To the best of our knowledge, this discriminative study of SZ is the first to combine multi-biological data of gut microbiota data, blood data, and EEG data. We developed an integrated framework of machine learning to discriminate patients with SZ from HCs. The main findings of this study are described below. (1) Using a combination of three types of biological features as input features for the classification, the best performance was achieved, with an accuracy of 91.7%, a sensitivity of 91.7%, a specificity of 91.7%, and an AUC of 96.5%. (2) the most discriminative features (top 5%) included gut microbiota features (*Lactobacillus*, *Haemophilus*, and *Prevotella)*, blood features (superoxide dismutase level, monocyte-lymphocyte ratio, and neutrophil count), and EEG features (nodal local efficiency, nodal efficiency, and nodal shortest path length in the temporal and frontal-parietal areas).

In this study, we developed an integrated framework of machine learning using a combination of multi-biological data, which is a promising direction for the identification of biomarkers for the diagnosis, prognosis, and treatment patients with SZ. The comparison of classification performance with existing studies is listed in Table 4. A recent study indicated that the diagnosis of SZ can be predicted with possible clinical utility by a computational machine learning algorithm using the combination of blood and cognitive biomarkers; more importantly, the integration of multi-biological data outperforms a single type of biological data^14^, consistent with our findings. Interestingly, an early SVM-based prediction of the later development of SZ in a familial high-risk cohort is possible and can be improved by combining schizotypal and neurocognitive features with neuroanatomical variables^33^. In summary, based on the integrated framework of machine learning, the combination of multi-biological data substantially improves the classification performance for patients with SZ. Our results revealed that the features from multiple biological datasets provided complementary information and can help to develop effective and objective biomarkers for the clinical diagnosis of SZ^1^.

**Table 4:** Comparison of classification performance with existing research.

References	Sample size	Input feature	Feature selection method	Classifier	Cross validation method	Performance
Shen et al.^4^	SZ = 64 HC = 53	Gut microbiota	Boruta variable selection	RF	None	AUC = 0.837
Brisa et al.^14^	SZ = 58 HC = 123	Blood and cognitive	PLS-DA	LDA	Tenfold	Accuracy = 0.86 AUC = 0.89
Jason et al.^20^	SZ = 40 HC = 12	EEG	None	SVM	None	Accuracy = 0.87 Sensitivity = 0.90 Specificity = 0.77
Sai Krishna Tikka et al.^21^	SZ = 38 HC = 20	EEG	None	SVM	Hold-out	Accuracy = 0.79 Sensitivity = 0.92 Specificity = 0.50 AUC = 0.71
Our best	SZ = 49 HC = 50	Gut microbiota Blood EEG	None	SVM	Tenfold	Accuracy = 0.92 Sensitivity = 0.92 Specificity = 0.92 AUC = 0.97

*RF* random forest, *PLS-DA* partial least squares discriminant analysis, *LDA* linear discriminant analysis, *EEG* electroencephalogram, *SVM* support vector machine, *AUC* area under the receiver operating characteristic curve.

To date, although numerous discriminative studies of SZ have used either data of blood-based^6,34,35^, or neuroimaging data^9,10,26,36,37^, few studies have investigated the potential of biomarkers for the diagnosis of SZ using gut microbiota data. Based on accumulating evidence, the gut microbiota bidirectionally communicates with the central nervous system through the microbiome-gut-brain axis (MGBA), thereby influencing brain function and behavior^38,39^. Recently, a few studies have focused on the role of the MGBA in SZ and revealed several alterations in the gut microbiota in patients with SZ^4,40–42^. These reports of an altered gut microbiotas are consistent with the finding from study, for which the most informative features of the gut microbiota include *Lactobacillus*, *Haemophilus*, *Collinsella*, *Clostridium,* and *Prevotella*. Furthermore, Yuan et al.^42^ have shown that changes in the gut microbiota and its metabolites may cause neuronal damage. *Lactobacillus* stimulate TNF production; therefore, *Lactobacillus* may induce changes in inflammatory factors that induce SZ^43^. On the other hand, short-chain fatty acids (SCFAs), the primary bacterial metabolites produced, can enter the central nervous system through the blood–brain barrier (BBB)^44^. *Clostridium* is the main source of propionate in the gut, indicating that *Clostridium* may influence the BBB and act on the brain by regulating SCFAs. In addition, *Collinsella* has been shown to produce the proinflammatory cytokine IL-17a and to alter intestinal permeability by promoting the release of neurotransmitters produced by gut microbiota^45^, thereby acting on the central nervous system. Above all, these investigations suggested that the gut microbiota may affect the central nervous system by acting on several pathways, providing a physiological basis for validating the use of the gut microbiota as a biomarker in the classification of the two groups.

Among the blood features we extracted, those that contributed the most to the classification included SOD level, MLR, MON count, NEU count, CRP level, WBC cunt and NLR, consistent with previous studies using conventional univariate statistical analysis. Numerous studies and increasing evidence suggest that the oxidative stress contributes to the pathogenesis of SZ, and abnormalities in antioxidant enzymes, including SOD activity, are frequently observed in patients diagnosed with SZ^46–48^. A previous study^49^ indicated that SOD activity remained lower in patients with SZ and may be an important indirect biomarker of oxidative stress in individuals with SZ. The present findings provide additional evidence of increased oxidative stress in patients with SZ. Blood inflammatory and immune system abnormalities in patients with SZ have been widely reported, which lead to an increase in levels of inflammatory markers. The NEU count was reported to be increased in patients with chronic SZ^50^. An increased MON count has also been reported in patients with chronic SZ^51,52^. Furthermore, a moderately increased CRP level in patients with SZ compared to HCs has been observed^53–55^. Subjects with SZ have significantly elevated WBC counts. The MLR and NLR have recently been used as indicators of inflammation, and predictors of cardiovascular disease, the leading cause of death in patients with SZ. A recent meta-analysis revealed a significant increase in the NLR in patients with SZ^56^. Elevated MLR and NLR have been observed in patients with SZ, suggesting an increased inflammatory response in individuals with SZ^50^. Our experimental results are consistent with these studies.

Table 3 shows that the EEG features with heavy weight are primarily derived from the delta and alpha2 frequency bands and partly from the beta and gamma frequency bands. Previous investigators observed increases in delta and theta waves, decreases in alpha waves and increases in beta and gamma waves in individuals with SZ^9,10,12,14,57,58^. Moreover, the most prominent change was in the spectral power of the delta wave, which may support the development of a biological marker for diagnosing patients with SZ^9,10,59^. In addition, among these EEG features, node attributes including nodal local efficiency (aNLe), nodal efficiency (aNe), nodal clustering coefficient (aNCp), and degree centrality (aDc), contributed most to classifying patients with SZ. EEG studies have shown a disruption in the small-world attributes of patients with SZ in the resting state, with a lower clustering coefficient and a longer shortest path length^60^. In addition, global and local efficiency are lower in patients with SZ than that in healthy people^61^. The most discriminative EEG features in Table 3 are primarily concentrated in the temporal lobe and partly in the frontal lobe. Abnormalities in temporal and frontal lobe function and structure have been widely reported in patients with SZ^62^ The frontal and temporal lobes are primarily associated with higher cognitive functions, among which the temporal lobe is associated with hearing and language functions, which have been confirmed by MRI studies^63^. These results are consistent with previous structural and functional neurological findings.

## Limitations

The present study has several limitations. First, since this study employed a cross-sectional design, we cannot infer causality. Some evidence suggests that immune-inflammatory markers are altered from the beginning of SZ, and researchers have broadly accepted that inflammation plays a causal role in SZ. However, from a diagnostic perspective, this finding is irrelevant. A specific marker must only discriminate between two conditions, regardless of whether it is a cause, consequence, or correlate of the pathophysiological process. Second, a significant difference in education years was observed between the two participant groups, although the results remained unchanged when this factor was included as a covariate. Third, the sample size was moderate. A larger independent sample is essential to examine the reproducibility of our findings.

## Conclusions

In conclusion, we developed an integrated framework of machine learning and used the combination of multi-biological data to discriminate patients with SZ from HCs, which substantially improved the classification performance. Based on our results, features from multiple biological datasets provide complementary information that aids in providing effective and objective biomarkers to inform the clinical diagnosis of SZ, and our framework is effective at conveying comprehensive and complementary information for the purpose of classification.

## Supplementary Information

Supplementary Tables.
