# Supplementary material for: An integrated machine learning framework for a discriminative analysis of schizophrenia using multi-biological data
Source: Sci Rep. 2021 Jul 19;11:14636. doi: 10.1038/s41598-021-94007-9 (PMC8290033; doi:10.1038/s41598-021-94007-9)
Supplement: Supplementary file 1 — Supplementary Tables. [file 41598_2021_94007_MOESM1_ESM.docx]

# An integrated machine learning framework for a discriminative analysis of schizophrenia using multi-biological data

Peng-fei Ke, BE ^1, 3, 5^; Dong-sheng Xiong, MS ^1, 3, 5^; Jia-hui Li, ME ^1, 3, 5^; Zhi-lin Pan, BE ^1, 3, 5^; Jing Zhou, PhD ^1, 3, 5^; Shi-jia Li, ME ^1, 3, 5^; Jie Song, MS ^1, 3, 5^; Xiao-yi Chen, BE ^1 3, 5^; Gui-xiang Li, ME ^4,^ ^7^; Jun Chen, ME ^4, 7^; Xiao-bo Li, PhD ^8^; Yu-ping Ning, PhD ^2, 3^; Feng-chun Wu, PhD ^2, 3^* and Kai Wu, PhD ^1, 2, 3, 4, 5, 6, 7, 8, 9^*

^1^ Department of Biomedical Engineering, School of Material Science and Engineering, South China University of Technology, Guangzhou 510006, China

^2^ The Affiliated Brain Hospital of Guangzhou Medical University, Guangzhou Huiai Hospital, Guangzhou 510370, China

^3^ Guangdong Engineering Technology Research Center for Translational Medicine of Mental Disorders, Guangzhou 510370, China

^4^ Guangdong Engineering Technology Research Center for Diagnosis and Rehabilitation of Dementia, Guangzhou 510500, China

^5^ National Engineering Research Center for Tissue Restoration and Reconstruction, South China University of Technology, Guangzhou 510006, China

^6^ Key Laboratory of Biomedical Engineering of Guangdong Province, South China University of Technology, Guangzhou 510006, China

^7^ National Engineering Research Center for Healthcare Devices, Guangzhou 510500, China

^8^ Department of Biomedical Engineering, New Jersey Institute of Technology, Newark, NJ, U.S

^9^ Department of Nuclear Medicine and Radiology, Institute of Development, Aging and Cancer, Tohoku University, Sendai 980-8575, Japan

* Address for correspondence:

Kai Wu, PhD.

School of Material Science

South China University of Technology

Guangzhou, Guangdong 510006, China

Tel: 86-135-8037-8315

Email: [kaiwu@scut.edu.cn](mailto:kaiwu@scut.edu.cn)

Fengchun Wu, Ph.D.

The Affiliated Brain Hospital of Guangzhou Medical University

Guangzhou Huiai Hospital

Guangzhou, Guangdong 510370, China

Tel: +86-020-8126-8212

Email: [13580380071@163.com](mailto:13580380071@163.com)

**Supplementary online content**

**eTable 1.** Classification Performance Including Accuracy, Sensitivity, Specificity, AUC and p Value under 10-Fold Cross Validation Method with Four Different Input Features, Three Feature Selection Algorithms and Five Classifiers.

**eTable 2**. Classification Performance Including Accuracy, Sensitivity, Specificity, AUC and *p* Value under 5-Fold Cross Validation Method with Four Different Input Features, Three Feature Selection Algorithms and Five Classifiers.

**eTable 3**. Classification Performance Including Accuracy, Sensitivity, Specificity, AUC and *p* Value under 3-Fold Cross Validation Method with Four Different Input Features, Three Feature Selection Algorithms and Five Classifiers.

**eTable 4**. Classification Performance Including Accuracy, Sensitivity, Specificity, AUC and *p* Value under Leave-One-Out Cross Validation Method with Four Different Input Features, Three Feature Selection Algorithms and Five Classifiers.

**eTable 1. Classification Performance Including Accuracy, Sensitivity, Specificity, AUC and p Value under 10-Fold Cross Validation Method with Four Different Input Features, Three Feature Selection Algorithms and Five Classifiers.**

| **Feature Selection method** | **Classifier** | **Accuracy (%)** | **Sensitivity (%)** | **Specificity (%)** | **AUC** | ***p***  **value^a^** |
| --- | --- | --- | --- | --- | --- | --- |
| **Input feature: Gut microbiota features (n=77)** | | | | | | |
| None^b^ | KNN | 41.7 | 41.7 | 41.7 | 0.50 | .21 |
|  | LR | 58.3 | 58.3 | 58.3 | 0.57 | .04 |
|  | LDA | 62.5 | 58.3 | 66.7 | 0.71 | .17 |
|  | RF | 75.0 | 66.7 | 83.3 | 0.82 | .11 |
|  | SVM | 54.2 | 50.0 | 58.3 | 0.46 | .13 |
| RFE | KNN | 50.0 | 33.3 | 66.7 | 0.48 | .010 |
|  | LR | 58.3 | 58.3 | 58.3 | 0.56 | .05 |
|  | LDA | 62.5 | 58.3 | 66.7 | 0.64 | .04 |
|  | RF | 70.8 | 58.3 | 83.3 | 0.80 | .03 |
|  | SVM | 54.2 | 50.0 | 58.3 | 0.47 | .11 |
| PCA | KNN | 50.0 | 58.3 | 41.7 | 0.46 | .05 |
|  | LR | 79.2 | 83.3 | 75.0 | 0.85 | .19 |
|  | LDA | 79.2 | 83.3 | 75.0 | 0.85 | .24 |
|  | RF | 66.7 | 66.7 | 66.7 | 0.72 | .14 |
|  | SVM | 70.8 | 75.0 | 66.7 | 0.79 | .28 |
| ANOVA | KNN | 62.5 | 50.0 | 75.0 | 0.67 | .05 |
|  | LR | 58.3 | 66.7 | 50.0 | 0.54 | .06 |
|  | LDA | 58.3 | 58.3 | 58.3 | 0.66 | .09 |
|  | RF | 62.5 | 58.3 | 66.7 | 0.70 | .010 |
|  | SVM | 66.7 | 66.7 | 66.7 | 0.77 | .04 |
| **Input feature: Blood features (n=12)** | | | | | | |
| None | KNN | 83.3 | 83.3 | 83.3 | 0.88 | .010 |
|  | LR | 75.0 | 66.7 | 83.3 | 0.85 | .010 |
|  | LDA | 75.0 | 75.0 | 75.0 | 0.88 | .010 |
|  | RF | 70.8 | 66.7 | 75.0 | 0.83 | .010 |
|  | SVM | 70.8 | 75.0 | 66.7 | 0.78 | .010 |
| RFE | KNN | NA^c^ | NA | NA | NA | NA |
|  | LR | NA | NA | NA | NA | NA |
|  | LDA | NA | NA | NA | NA | NA |
|  | RF | NA | NA | NA | NA | NA |
|  | SVM | NA | NA | NA | NA | NA |
| PCA | KNN | NA | NA | NA | NA | NA |
|  | LR | NA | NA | NA | NA | NA |
|  | LDA | NA | NA | NA | NA | NA |
|  | RF | NA | NA | NA | NA | NA |
|  | SVM | NA | NA | NA | NA | NA |
| **Feature Selection method** | **Classifier** | **Accuracy (%)** | **Sensitivity (%)** | **Specificity (%)** | **AUC** | ***p***  **value^a^** |
| ANOVA | KNN | 62.5 | 58.3 | 66.7 | 0.65 | .010 |
|  | LR | 62.5 | 58.3 | 66.7 | 0.70 | .010 |
|  | LDA | 62.5 | 58.3 | 66.7 | 0.70 | .010 |
|  | RF | 62.5 | 58.3 | 66.7 | 0.58 | .010 |
|  | SVM | 62.5 | 66.7 | 58.3 | 0.70 | .010 |
| **Input feature: EEG features (n=574)** | | | | | | |
| None | KNN | 66.7 | 66.7 | 66.7 | 0.76 | .06 |
|  | LR | 75.0 | 75.0 | 75.0 | 0.86 | .09 |
|  | LDA | 75.0 | 75.0 | 75.0 | 0.80 | .12 |
|  | RF | 79.2 | 83.3 | 75.0 | 0.88 | .02 |
|  | SVM | 70.8 | 75.0 | 66.7 | 0.84 | .02 |
| RFE | KNN | 75.0 | 75.0 | 75.0 | 0.80 | .010 |
|  | LR | 79.2 | 83.3 | 75.0 | 0.89 | .07 |
|  | LDA | 66.7 | 66.7 | 66.7 | 0.79 | .05 |
|  | RF | 79.2 | 83.3 | 75.0 | 0.90 | .010 |
|  | SVM | 75.0 | 75.0 | 75.0 | 0.84 | .04 |
| PCA | KNN | 70.8 | 83.3 | 58.3 | 0.68 | .05 |
|  | LR | 83.3 | 83.3 | 83.3 | 0.86 | .010 |
|  | LDA | 75.0 | 83.3 | 66.7 | 0.81 | .13 |
|  | RF | 70.8 | 75.0 | 66.7 | 0.72 | .11 |
|  | SVM | 83.3 | 83.3 | 83.3 | 0.85 | .07 |
| ANOVA | KNN | 70.8 | 75.0 | 66.7 | 0.74 | .03 |
|  | LR | 75.0 | 75.0 | 75.0 | 0.85 | .03 |
|  | LDA | 75.0 | 75.0 | 75.0 | 0.85 | .06 |
|  | RF | 70.8 | 83.3 | 59.3 | 0.82 | .010 |
|  | SVM | 79.2 | 83.3 | 75.0 | 0.85 | .010 |
| **Input feature: Combined features (n=663)** | | | | | | |
| None | KNN | 79.2 | 83.3 | 75.0 | 0.85 | .02 |
|  | LR | 91.7 | 91.7 | 91.7 | 0.95 | .010 |
|  | LDA | 79.2 | 75.0 | 83.3 | 0.86 | .13 |
|  | RF | 83.3 | 91.7 | 75.0 | 0.94 | .010 |
|  | SVM | 91.7 | 91.7 | 91.7 | 0.97 | .010 |
| RFE | KNN | 79.2 | 83.3 | 75.0 | 0.91 | .010 |
|  | LR | 75.0 | 66.7 | 83.3 | 0.84 | .010 |
|  | LDA | 75.0 | 75.0 | 75.0 | 0.83 | .010 |
|  | RF | 75.0 | 75.0 | 75.0 | 0.76 | .010 |
|  | SVM | 79.2 | 75.0 | 83.3 | 0.88 | .010 |
| PCA | KNN | 79.2 | 75.0 | 83.3 | 0.82 | .010 |
|  | LR | 79.2 | 75.0 | 83.3 | 0.88 | .010 |
|  | LDA | 87.5 | 83.3 | 91.7 | 0.88 | .010 |
|  | RF | 66.7 | 75.0 | 58.3 | 0.69 | .010 |
| **Feature Selection method** | **Classifier** | **Accuracy (%)** | **Sensitivity (%)** | **Specificity (%)** | **AUC** | ***p***  **value^a^** |
| PCA | SVM | 75.0 | 75.0 | 75.0 | 0.84 | .02 |
| ANOVA | KNN | 70.8 | 58.3 | 83.3 | 0.84 | .010 |
|  | LR | 66.7 | 58.3 | 75.0 | 0.79 | .010 |
|  | LDA | 62.5 | 58.3 | 66.7 | 0.74 | .010 |
|  | RF | 75.0 | 66.7 | 83.3 | 0.87 | .010 |
|  | SVM | 54.1 | 58.3 | 50.0 | 0.74 | .010 |
| Abbreviations: AUC, area under the receiver operating characteristic curve; RFE, recursive feature elimination; PCA, principal component analysis; ANOVA, analysis of variance; KNN, k-nearest neighbor; LR, logistic regression; LDA, linear discriminant analysis; RF, random forest; SVM, support vector machine; EEG, Electroencephalogram; NA, not available.   1. The statistical significance of permutation test is set at p < 0.05 2. None means no feature selection algorithm is used. 3. Due to the small number of blood features, RFE and PCA feature selection algorithms cannot be used. | | | | | | |

**eTable 2. Classification Performance Including Accuracy, Sensitivity, Specificity, AUC and p Value under 5-Fold Cross Validation Method with Four Different Input Features, Three Feature Selection Algorithms and Five Classifiers.**

| **Feature Selection method** | **Classifier** | **Accuracy (%)** | **Sensitivity (%)** | **Specificity (%)** | **AUC** | ***p***  **value^a^** |
| --- | --- | --- | --- | --- | --- | --- |
| **Input feature: Gut microbiota features (n=77)** | | | | | | |
| None^b^ | KNN | 41.7 | 25.0 | 58.3 | 0.51 | .09 |
|  | LR | 58.3 | 58.3 | 58.3 | 0.56 | .64 |
|  | LDA | 58.3 | 58.3 | 58.3 | 0.58 | .22 |
|  | RF | 75.0 | 66.7 | 83.3 | 0.82 | .08 |
|  | SVM | 66.7 | 66.7 | 66.7 | 0.22 | .59 |
| RFE | KNN | 58.3 | 41.7 | 75.0 | 0.69 | .02 |
|  | LR | 58.3 | 58.3 | 58.3 | 0.56 | .46 |
|  | LDA | 45.8 | 33.3 | 58.3 | 0.52 | .04 |
|  | RF | 66.7 | 66.7 | 66.7 | 0.79 | .05 |
|  | SVM | 54.2 | 16.7 | 91.7 | 0.34 | .11 |
| PCA | KNN | 41.7 | 50.0 | 33.3 | 0.38 | .16 |
|  | LR | 79.2 | 91.7 | 66.7 | 0.86 | .37 |
|  | LDA | 75.0 | 75.0 | 75.0 | 0.82 | .50 |
|  | RF | 70.8 | 66.7 | 75.0 | 0.62 | .06 |
|  | SVM | 70.8 | 75.0 | 66.7 | 0.80 | .24 |
| ANOVA | KNN | 58.3 | 58.3 | 58.3 | 0.60 | .04 |
|  | LR | 66.7 | 66.7 | 66.7 | 0.71 | .11 |
|  | LDA | 41.7 | 41.7 | 41.7 | 0.40 | .02 |
|  | RF | 83.3 | 75.0 | 91.7 | 0.94 | .04 |
|  | SVM | 66.7 | 75.0 | 58.3 | 0.78 | .11 |
| **Input feature: Blood features (n=12)** | | | | | | |
| None | KNN | 83.3 | 83.3 | 83.3 | 0.88 | .010 |
|  | LR | 75.0 | 66.7 | 83.3 | 0.87 | .010 |
|  | LDA | 75.0 | 75.0 | 75.0 | 0.88 | .010 |
|  | RF | 70.8 | 66.7 | 75.0 | 0.83 | .010 |
|  | SVM | 70.8 | 75.0 | 66.7 | 0.78 | .010 |
| RFE | KNN | NA^c^ | NA | NA | NA | NA |
|  | LR | NA | NA | NA | NA | NA |
|  | LDA | NA | NA | NA | NA | NA |
|  | RF | NA | NA | NA | NA | NA |
|  | SVM | NA | NA | NA | NA | NA |
| PCA | KNN | NA | NA | NA | NA | NA |
|  | LR | NA | NA | NA | NA | NA |
|  | LDA | NA | NA | NA | NA | NA |
|  | RF | NA | NA | NA | NA | NA |
|  | SVM | NA | NA | NA | NA | NA |
| **Feature Selection method** | **Classifier** | **Accuracy (%)** | **Sensitivity (%)** | **Specificity (%)** | **AUC** | ***p***  **value^a^** |
| ANOVA | KNN | 62.5 | 58.3 | 66.7 | 0.61 | .010 |
|  | LR | 62.5 | 58.3 | 66.7 | 0.70 | .010 |
|  | LDA | 62.5 | 58.3 | 66.7 | 0.70 | .010 |
|  | RF | 62.5 | 58.3 | 66.7 | 0.71 | .010 |
|  | SVM | 62.5 | 66.7 | 58.3 | 0.70 | .010 |
| **Input feature: EEG features (n=574)** | | | | | | |
| None | KNN | 62.5 | 66.7 | 58.3 | 0.69 | .04 |
|  | LR | 79.2 | 83.3 | 75.0 | 0.87 | .03 |
|  | LDA | 75.0 | 75.0 | 75.0 | 0.80 | .03 |
|  | RF | 79.2 | 83.3 | 75.0 | 0.88 | .04 |
|  | SVM | 70.8 | 75.0 | 66.7 | 0.84 | .08 |
| RFE | KNN | 79.2 | 83.3 | 75.0 | 0.82 | .04 |
|  | LR | 79.2 | 83.3 | 75.0 | 0.88 | .010 |
|  | LDA | 70.8 | 66.7 | 75.0 | 0.80 | .02 |
|  | RF | 79.2 | 91.7 | 66.7 | 0.82 | .010 |
|  | SVM | 70.8 | 75.0 | 66.7 | 0.85 | .08 |
| PCA | KNN | 70.8 | 66.7 | 75.0 | 0.74 | .02 |
|  | LR | 83.3 | 83.3 | 83.3 | 0.86 | .010 |
|  | LDA | 79.2 | 83.3 | 75.0 | 0.86 | .06 |
|  | RF | 79.2 | 83.3 | 75.0 | 0.82 | .03 |
|  | SVM | 83.3 | 83.3 | 83.3 | 0.90 | .02 |
| ANOVA | KNN | 75.0 | 75.0 | 75.0 | 0.78 | .04 |
|  | LR | 79.2 | 83.3 | 75.0 | 0.84 | .010 |
|  | LDA | 79.2 | 83.3 | 75.0 | 0.83 | .03 |
|  | RF | 70.8 | 83.3 | 58.3 | 0.89 | .010 |
|  | SVM | 75.0 | 75.0 | 75.0 | 0.83 | .010 |
| **Input feature: Combined features (n=663)** | | | | | | |
| None | KNN | 70.8 | 75.0 | 66.7 | 0.79 | .04 |
|  | LR | 87.5 | 83.3 | 91.7 | 0.92 | .010 |
|  | LDA | 75.0 | 66.7 | 83.3 | 0.82 | .02 |
|  | RF | 79.2 | 75.0 | 83.3 | 0.93 | .010 |
|  | SVM | 91.7 | 91.7 | 91.7 | 0.92 | .010 |
| RFE | KNN | 70.8 | 75.0 | 66.7 | 0.78 | .010 |
|  | LR | 62.5 | 66.7 | 58.3 | 0.76 | .010 |
|  | LDA | 58.3 | 58.3 | 58.3 | 0.77 | .010 |
|  | RF | 62.5 | 58.3 | 66.7 | 0.81 | .010 |
|  | SVM | 50.0 | 50.0 | 50.0 | 0.69 | .010 |
| PCA | KNN | 62.5 | 50.0 | 75.0 | 0.74 | .010 |
|  | LR | 87.5 | 83.3 | 91.7 | 0.90 | .010 |
|  | LDA | 79.2 | 75.0 | 83.3 | 0.88 | .010 |
|  | RF | 66.7 | 75.0 | 58.3 | 0.81 | .020 |
| **Feature Selection method** | **Classifier** | **Accuracy (%)** | **Sensitivity (%)** | **Specificity (%)** | **AUC** | ***p***  **value^a^** |
| PCA | SVM | 79.2 | 75.0 | 83.3 | 0.88 | .010 |
| ANOVA | KNN | 70.8 | 58.3 | 83.3 | 0.85 | .010 |
|  | LR | 70.8 | 66.7 | 75.0 | 0.88 | .010 |
|  | LDA | 50.0 | 41.7 | 58.3 | 0.54 | .010 |
|  | RF | 83.3 | 83.3 | 83.3 | 0.88 | .010 |
|  | SVM | 75.0 | 66.7 | 83.3 | 0.81 | .010 |
| Abbreviations: AUC, area under the receiver operating characteristic curve; RFE, recursive feature elimination; PCA, principal component analysis; ANOVA, analysis of variance; KNN, k-nearest neighbor; LR, logistic regression; LDA, linear discriminant analysis; RF, random forest; SVM, support vector machine; EEG, Electroencephalogram; NA, not available.   1. The statistical significance of permutation test is set at p < 0.05 2. None means no feature selection algorithm is used. 3. Due to the small number of blood features, RFE and PCA feature selection algorithms cannot be used. | | | | | | |

**eTable 3. Classification Performance Including Accuracy, Sensitivity, Specificity, AUC and *p* Value under 5-Fold Cross Validation Method with Four Different Input Features, Three Feature Selection Algorithms and Five Classifiers.**

| **Feature Selection method** | **Classifier** | **Accuracy (%)** | **Sensitivity (%)** | **Specificity (%)** | **AUC** | ***p***  **value^a^** |
| --- | --- | --- | --- | --- | --- | --- |
| **Input feature: Gut microbiota features (n=77)** | | | | | | |
| None^b^ | KNN | 54.2 | 25.0 | 83.3 | 0.54 | .57 |
|  | LR | 54.2 | 50.0 | 58.3 | 0.55 | .72 |
|  | LDA | 58.3 | 58.3 | 58.3 | 0.58 | .07 |
|  | RF | 75.0 | 66.7 | 83.3 | 0.82 | .82 |
|  | SVM | 54.2 | 50.0 | 58.3 | 0.50 | .53 |
| RFE | KNN | 50.0 | 41.7 | 58.3 | 0.55 | .15 |
|  | LR | 54.2 | 50.0 | 58.3 | 0.60 | .61 |
|  | LDA | 41.7 | 41.7 | 41.7 | 0.41 | .07 |
|  | RF | 79.2 | 75.0 | 83.3 | 0.93 | .45 |
|  | SVM | 62.5 | 33.3 | 91.7 | 0.40 | .24 |
| PCA | KNN | 50.0 | 50.0 | 50.0 | 0.45 | .06 |
|  | LR | 79.2 | 75.0 | 83.3 | 0.83 | .88 |
|  | LDA | 83.3 | 83.3 | 83.3 | 0.86 | .98 |
|  | RF | 66.7 | 66.7 | 66.7 | 0.72 | .57 |
|  | SVM | 58.3 | 25.0 | 91.7 | 0.25 | .80 |
| ANOVA | KNN | 58.3 | 16.7 | 100.0 | 0.63 | .30 |
|  | LR | 54.2 | 50.0 | 58.3 | 0.55 | .72 |
|  | LDA | 58.3 | 58.3 | 58.3 | 0.62 | .03 |
|  | RF | 75.0 | 66.7 | 83.3 | 0.82 | .25 |
|  | SVM | 58.3 | 66.7 | 50.0 | 0.64 | .32 |
| **Input feature: Blood features (n=12)** | | | | | | |
| None | KNN | 79.2 | 75.0 | 83.3 | 0.86 | .010 |
|  | LR | 75.0 | 66.7 | 83.3 | 0.87 | .010 |
|  | LDA | 75.0 | 75.0 | 75.0 | 0.88 | .010 |
|  | RF | 70.8 | 66.7 | 75.0 | 0.83 | .010 |
|  | SVM | 70.8 | 75.0 | 66.7 | 0.78 | .010 |
| RFE | KNN | NA^c^ | NA | NA | NA | NA |
|  | LR | NA | NA | NA | NA | NA |
|  | LDA | NA | NA | NA | NA | NA |
|  | RF | NA | NA | NA | NA | NA |
|  | SVM | NA | NA | NA | NA | NA |
| PCA | KNN | NA | NA | NA | NA | NA |
|  | LR | NA | NA | NA | NA | NA |
|  | LDA | NA | NA | NA | NA | NA |
|  | RF | NA | NA | NA | NA | NA |
|  | SVM | NA | NA | NA | NA | NA |
| **Feature Selection method** | **Classifier** | **Accuracy (%)** | **Sensitivity (%)** | **Specificity (%)** | **AUC** | ***p***  **value^a^** |
| ANOVA | KNN | 70.8 | 66.7 | 75.0 | 0.72 | .010 |
|  | LR | 75.0 | 75.0 | 75.0 | 0.83 | .010 |
|  | LDA | 62.5 | 58.3 | 66.7 | 0.70 | .010 |
|  | RF | 62.5 | 58.3 | 66.7 | 0.58 | .010 |
|  | SVM | 62.5 | 66.7 | 58.3 | 0.70 | .010 |
| **Input feature: EEG features (n=574)** | | | | | | |
| None | KNN | 70.8 | 75.0 | 66.7 | 0.73 | .19 |
|  | LR | 79.2 | 83.3 | 75.0 | 0.88 | .03 |
|  | LDA | 75.0 | 75.0 | 75.0 | 0.80 | .08 |
|  | RF | 79.2 | 83.3 | 75.0 | 0.88 | .010 |
|  | SVM | 70.8 | 75.0 | 66.7 | 0.85 | .16 |
| RFE | KNN | 66.7 | 83.3 | 50.0 | 0.78 | .010 |
|  | LR | 79.2 | 83.3 | 75.0 | 0.88 | .03 |
|  | LDA | 87.5 | 83.3 | 91.7 | 0.88 | .010 |
|  | RF | 70.8 | 83.3 | 58.3 | 0.85 | .010 |
|  | SVM | 70.8 | 75.0 | 66.7 | 0.84 | .03 |
| PCA | KNN | 66.7 | 75.0 | 58.3 | 0.68 | .010 |
|  | LR | 83.3 | 83.3 | 83.3 | 0.86 | .010 |
|  | LDA | 75.0 | 83.3 | 66.7 | 0.81 | .23 |
|  | RF | 75.0 | 91.7 | 58.3 | 0.77 | .03 |
|  | SVM | 83.3 | 83.3 | 83.3 | 0.90 | .02 |
| ANOVA | KNN | 75.0 | 83.3 | 66.7 | 0.76 | .010 |
|  | LR | 79.2 | 83.3 | 75.0 | 0.84 | .010 |
|  | LDA | 75.0 | 75.0 | 75.0 | 0.79 | .010 |
|  | RF | 62.5 | 66.7 | 58.3 | 0.77 | .010 |
|  | SVM | 75.0 | 75.0 | 75.0 | 0.83 | .02 |
| **Input feature: Combined features (n=663)** | | | | | | |
| None | KNN | 70.8 | 58.3 | 83.3 | 0.75 | .04 |
|  | LR | 87.5 | 83.3 | 91.7 | 0.92 | .010 |
|  | LDA | 75.0 | 66.7 | 83.3 | 0.82 | .29 |
|  | RF | 79.2 | 75.0 | 83.3 | 0.93 | .010 |
|  | SVM | 91.7 | 91.7 | 91.7 | 0.92 | .010 |
| RFE | KNN | 66.7 | 66.7 | 66.7 | 0.75 | .010 |
|  | LR | 62.5 | 66.7 | 58.3 | 0.79 | .010 |
|  | LDA | 58.3 | 58.3 | 58.3 | 0.77 | .010 |
|  | RF | 62.5 | 58.3 | 66.7 | 0.81 | .010 |
|  | SVM | 66.7 | 66.7 | 66.7 | 0.79 | .010 |
| PCA | KNN | 70.8 | 66.7 | 75.0 | 0.77 | .06 |
|  | LR | 87.5 | 83.3 | 91.7 | 0.90 | .02 |
|  | LDA | 79.2 | 75.0 | 83.3 | 0.88 | .05 |
|  | RF | 66.7 | 75.0 | 58.3 | 0.79 | .08 |
| **Feature Selection method** | **Classifier** | **Accuracy (%)** | **Sensitivity (%)** | **Specificity (%)** | **AUC** | ***p***  **value^a^** |
| PCA | SVM | 79.2 | 83.3 | 75.0 | 0.86 | .010 |
| ANOVA | KNN | 70.8 | 58.3 | 83.3 | 0.88 | .010 |
|  | LR | 70.8 | 58.3 | 83.3 | 0.86 | .010 |
|  | LDA | 50.0 | 41.7 | 58.3 | 0.54 | .010 |
|  | RF | 70.8 | 66.7 | 75.0 | 0.83 | .010 |
|  | SVM | 75.0 | 66.7 | 83.3 | 0.81 | .010 |
| Abbreviations: AUC, area under the receiver operating characteristic curve; RFE, recursive feature elimination; PCA, principal component analysis; ANOVA, analysis of variance; KNN, k-nearest neighbor; LR, logistic regression; LDA, linear discriminant analysis; RF, random forest; SVM, support vector machine; EEG, Electroencephalogram; NA, not available.   1. The statistical significance of permutation test is set at p < 0.05 2. None means no feature selection algorithm is used. 3. Due to the small number of blood features, RFE and PCA feature selection algorithms cannot be used | | | | | | |

**eTable 4. Classification Performance Including Accuracy, Sensitivity, Specificity, AUC and *p* Value under Leave-One-Out Cross Validation Method with Four Different Input Features, Three Feature Selection Algorithms and Five Classifiers.**

| **Feature Selection method** | **Classifier** | **Accuracy (%)** | **Sensitivity (%)** | **Specificity (%)** | **AUC** | ***p***  **value^a^** |
| --- | --- | --- | --- | --- | --- | --- |
| **Input feature: Gut microbiota features (n=77)** | | | | | | |
| None^b^ | KNN | 41.7 | 41.7 | 41.7 | 0.50 | .09 |
|  | LR | 54.2 | 50.0 | 58.3 | 0.54 | .08 |
|  | LDA | 62.5 | 58.3 | 66.7 | 0.71 | .33 |
|  | RF | 70.8 | 58.3 | 83.3 | 0.77 | .09 |
|  | SVM | 54.2 | 50.0 | 58.3 | 0.46 | .06 |
| RFE | KNN | 45.8 | 33.3 | 58.3 | 0.50 | .02 |
|  | LR | 50.0 | 50.0 | 50.0 | 0.49 | .03 |
|  | LDA | 41.7 | 41.7 | 41.7 | 0.41 | .03 |
|  | RF | 62.5 | 58.3 | 66.7 | 0.67 | .03 |
|  | SVM | 58.3 | 58.3 | 58.3 | 0.49 | .03 |
| PCA | KNN | 50.0 | 58.3 | 41.7 | 0.49 | .14 |
|  | LR | 79.2 | 91.7 | 66.7 | 0.86 | .26 |
|  | LDA | 83.3 | 83.3 | 83.3 | 0.86 | .09 |
|  | RF | 66.7 | 66.7 | 66.7 | 0.72 | .07 |
|  | SVM | 75.0 | 91.7 | 58.3 | 0.87 | .06 |
| ANOVA | KNN | 41.7 | 33.3 | 50.0 | 0.47 | .02 |
|  | LR | 54.2 | 50.0 | 58.3 | 0.54 | .08 |
|  | LDA | 45.8 | 50.0 | 41.7 | 0.50 | .05 |
|  | RF | 83.3 | 75.0 | 91.7 | 0.94 | .010 |
|  | SVM | 58.3 | 66.7 | 50.0 | 0.68 | .010 |
| **Input feature: Blood features (n=12)** | | | | | | |
| None | KNN | 83.3 | 83.3 | 83.3 | 0.86 | .010 |
|  | LR | 75.0 | 66.7 | 83.3 | 0.85 | .010 |
|  | LDA | 75.0 | 75.0 | 75.0 | 0.88 | .010 |
|  | RF | 75.0 | 75.0 | 75.0 | 0.84 | .010 |
|  | SVM | 70.8 | 75.0 | 66.7 | 0.78 | .010 |
| RFE | KNN | NA^c^ | NA | NA | NA | NA |
|  | LR | NA | NA | NA | NA | NA |
|  | LDA | NA | NA | NA | NA | NA |
|  | RF | NA | NA | NA | NA | NA |
|  | SVM | NA | NA | NA | NA | NA |
| PCA | KNN | NA | NA | NA | NA | NA |
|  | LR | NA | NA | NA | NA | NA |
|  | LDA | NA | NA | NA | NA | NA |
|  | RF | NA | NA | NA | NA | NA |
|  | SVM | NA | NA | NA | NA | NA |
| **Feature Selection method** | **Classifier** | **Accuracy (%)** | **Sensitivity (%)** | **Specificity (%)** | **AUC** | ***p***  **value^a^** |
|  |  |  |  |  |  |  |
| ANOVA | KNN | 75.0 | 83.3 | 66.7 | 0.84 | .010 |
|  | LR | 62.5 | 58.3 | 66.7 | 0.70 | .010 |
|  | LDA | 62.5 | 58.3 | 66.7 | 0.70 | .010 |
|  | RF | 66.7 | 58.3 | 75.0 | 0.73 | .010 |
|  | SVM | 62.5 | 66.7 | 58.3 | 0.70 | .010 |
| **Input feature: EEG features (n=574)** | | | | | | |
| None | KNN | 62.5 | 66.7 | 58.3 | 0.79 | .06 |
|  | LR | 79.2 | 83.3 | 75.0 | 0.88 | .05 |
|  | LDA | 75.0 | 75.0 | 75.0 | 0.80 | .19 |
|  | RF | 79.2 | 83.3 | 75.0 | 0.88 | .08 |
|  | SVM | 70.8 | 75.0 | 66.7 | 0.84 | .07 |
| RFE | KNN | 79.2 | 83.3 | 75.0 | 0.80 | .02 |
|  | LR | 79.2 | 83.3 | 75.0 | 0.87 | .04 |
|  | LDA | 91.7 | 91.7 | 91.7 | 0.93 | .08 |
|  | RF | 70.8 | 83.3 | 58.3 | 0.88 | .010 |
|  | SVM | 70.8 | 75.0 | 66.7 | 0.84 | .04 |
| PCA | KNN | 66.7 | 66.7 | 66.7 | 0.73 | .05 |
|  | LR | 83.3 | 83.3 | 83.3 | 0.86 | .03 |
|  | LDA | 79.2 | 83.3 | 75.0 | 0.86 | .02 |
|  | RF | 70.8 | 83.3 | 58.3 | 0.87 | .20 |
|  | SVM | 83.3 | 83.3 | 83.3 | 0.85 | .04 |
| ANOVA | KNN | 70.8 | 75.0 | 66.7 | 0.72 | .010 |
|  | LR | 79.2 | 83.3 | 75.0 | 0.84 | .010 |
|  | LDA | 75.0 | 75.0 | 75.0 | 0.79 | .010 |
|  | RF | 70.8 | 58.3 | 83.3 | 0.82 | .02 |
|  | SVM | 79.2 | 83.3 | 75.0 | 0.79 | .03 |
| **Input feature: Combined features (n=663)** | | | | | | |
| None | KNN | 75.0 | 66.7 | 83.3 | 0.81 | .02 |
|  | LR | 87.5 | 83.3 | 91.7 | 0.92 | .010 |
|  | LDA | 75.0 | 66.7 | 83.3 | 0.82 | .02 |
|  | RF | 79.2 | 75.0 | 83.3 | 0.94 | .010 |
|  | SVM | 87.5 | 91.7 | 83.3 | 0.94 | .010 |
| RFE | KNN | 79.2 | 83.3 | 75.0 | 0.86 | .010 |
|  | LR | 62.5 | 66.7 | 58.3 | 0.79 | .010 |
|  | LDA | 58.3 | 58.3 | 58.3 | 0.77 | .010 |
|  | RF | 62.5 | 58.3 | 66.7 | 0.81 | .010 |
|  | SVM | 83.3 | 83.3 | 83.3 | 0.88 | .010 |
| PCA | KNN | 79.2 | 83.3 | 75.0 | 0.85 | .02 |
|  | LR | 87.5 | 83.3 | 91.7 | 0.87 | .010 |
|  | LDA | 79.2 | 75.0 | 83.3 | 0.88 | .010 |
| **Feature Selection method** | **Classifier** | **Accuracy (%)** | **Sensitivity (%)** | **Specificity (%)** | **AUC** | ***p***  **value^a^** |
| PCA | RF | 70.8 | 83.3 | 58.3 | 0.74 | .09 |
|  | SVM | 79.2 | 91.7 | 66.7 | 0.81 | .19 |
| ANOVA | KNN | 70.8 | 58.3 | 83.3 | 0.88 | .010 |
|  | LR | 70.8 | 58.3 | 83.3 | 0.86 | .010 |
|  | LDA | 62.5 | 58.3 | 66.7 | 0.76 | .010 |
|  | RF | 70.8 | 66.7 | 75.0 | 0.82 | .010 |
|  | SVM | 70.8 | 58.3 | 83.3 | 0.81 | .010 |
| Abbreviations: AUC, area under the receiver operating characteristic curve; RFE, recursive feature elimination; PCA, principal component analysis; ANOVA, analysis of variance; KNN, k-nearest neighbor; LR, logistic regression; LDA, linear discriminant analysis; RF, random forest; SVM, support vector machine; EEG, Electroencephalogram; NA, not available.   1. The statistical significance of permutation test is set at p < 0.05. 2. None means no feature selection algorithm is used. 3. Due to the small number of blood features, RFE and PCA feature selection algorithms cannot be used. | | | | | | |
